# *Mycobacterium tuberculosis* resistance prediction and lineage classification from genome sequencing: comparison of automated analysis tools

**DOI:** 10.1038/srep46327

**Published:** 2017-04-20

**Authors:** Viola Schleusener, Claudio U. Köser, Patrick Beckert, Stefan Niemann, Silke Feuerriegel

**Affiliations:** 1 Division of Molecular and Experimental Mycobacteriology Group, Research Center Borstel, Borstel, Germany; 2 Department of Genetics, University of Cambridge, Cambridge, United Kingdom; 3 German Center for Infection Research, Borstel Site, Borstel Germany

**Keywords:** Bacterial genetics, Molecular medicine

## Abstract

Whole-genome sequencing (WGS) has the potential to accelerate drug-susceptibility testing (DST) to design appropriate regimens for drug-resistant tuberculosis (TB). Several recently developed automated software tools promise to standardize the analysis and interpretation of WGS data. We assessed five tools (CASTB, KvarQ, Mykrobe Predictor TB, PhyResSE, and TBProfiler) with regards to DST and phylogenetic lineage classification, which we compared with phenotypic DST, Sanger sequencing, and traditional typing results for a collection of 91 strains. The lineage classifications by the tools generally only differed in the resolution of the results. However, some strains could not be classified at all and one strain was misclassified. The sensitivities and specificities for isoniazid and rifampicin resistance of the tools were high, whereas the results for ethambutol, pyrazinamide, and streptomycin resistance were more variable. False-susceptible DST results were mainly due to missing mutations in the resistance catalogues that the respective tools employed for data interpretation. Notably, we also found cases of false-resistance because of the misclassification of polymorphisms as resistance mutations. In conclusion, the performance of current WGS analysis tools for DST is highly variable. Sustainable business models and a shared, high-quality catalogue of resistance mutations are needed to ensure the clinical utility of these tools.

**Supplementary information:**

The online version of this article (doi:10.1038/srep46327) contains supplementary material, which is available to authorized users.

## Introduction

Tuberculosis (TB) control is threatened by the emergence and spread of resistant Mycobacterium tuberculosis complex (MTBC) strains. In 2015, an estimated 480,000 new cases of multidrug-resistant (MDR, defined as resistant to the most effective first-line drugs isoniazid [INH] and rifampicin [RMP]) TB occurred globally. Of those, approximately 9.5% are extensively drug-resistant (XDR) with additional resistances to at least one fluoroquinolone and one injectable drug^1^. MDR and XDR TB requires prolonged treatment with more expensive and less effective drugs that can result in severe side effects^2^.

This situation is exacerbated by the fact that conventional, phenotypic drug-susceptibility testing (DST) can take weeks or months because of the slow growth rate of MTBC^3^. Targeted genotypic assays, such as line probe assays or the Cepheid Xpert MTB/RIF, have been developed to provide DST results within hours or days^4^. However, these assays only target the most frequent resistance mutations for a limited number of drugs.

By contrast, whole-genome sequencing (WGS) can theoretically be used to rule-in resistance to all drugs simultaneously, provided that the genetic basis of resistance is elucidated further (WGS directly from the primary sample would be ideal, but is not feasible reliably and cheaply at the moment)^5,6,7^. Yet, given the amount and complexity of WGS data, its interpretation represents a significant challenge. Specifically, three main steps have to be performed accurately. First, variants (i.e. SNPs, insertions or deletions) have to be identified accurately. Second, these variants have to be interpreted correctly (e.g. mutations that do not confer resistance have to be distinguished from mutations that do)^8^. Finally, a plain language report that contains clear and appropriate results has to be generated.

To date, three web-servers (CASTB, PhyResSE, and TBProfiler) and two software solutions (KvarQ and Mykrobe Predictor TB) have been developed to enable non-specialists to infer drug resistance from WGS data^9,10,11,12,13^. Moreover, all tools provide epidemiological typing results, which might be used for contact tracing or surveillance, and can affect patient treatment directly if a member of MTBC is identified that is intrinsically resistant to one or more TB drug^14^. The respective strengths and weaknesses of all of these tools have not been compared so far^15^. Using a collection of 91 clinical strains with resistance mutations that were confirmed by classical Sanger sequencing, we therefore set out to assess the performance and functionalities of these tools.

## Methods

### Evaluation dataset

Our collection consisted of 91 strains from Sierra Leone for which phenotypic DST results, WGS data (ENA accession number: PRJEB7727), Sanger sequencing data for key resistance genes (*katG, rpoB, embB, rrs, rpsL, gidB, pncA*, and, in some cases, *inhA, ahpC, embA*, and *embC*), as well as conventional typing data were available (Supplementary Tables S1 and S2). This set of strains has been described before^16,17^. Half of them were pan-susceptible, whereas the remaining strains were resistant to at least one antibiotic. The strains were highly diverse phylogenetically and comprised both *M. africanum* genotypes (West African I, n = 6; West African II, n = 14) and ten *M. tuberculosis* lineages (Beijing, n = 4; Cameroon, n = 4; EAI, n = 4; Ghana, n = 1; Haarlem, n = 11; LAM, n = 15; S-type, n = 3; Sierra Leone I, n = 7; Sierra Leone II, n = 9; X-type, n = 2). Using conventional methods (MIRU-VNTR, IS*6110*-RFLP, and spoligotyping), the lineage could not be identified for 11 strains. DNA was extracted from cultures as previously described and prepared for sequencing on an Illumina MiSeq benchtop sequencer (251 bp or 301 bp with paired-ends) using the Nextera XT kit according to the manufacturer’s instruction.

Moreover, we analysed three *M. bovis* genomes to investigate the ability of CASTB to identify intrinsic pyrazinamide (PZA) resistance (4258-00 (ENA accession: ERS457842), 751-01 (ERS458720), and 7540-01 (ERS458073))^8^.

### Software tools

#### CASTB version 1.1

CASTB is an automated analysis web-server for WGS data from MTBC strains (http:\\castb.ri.ncgm.go.jp\CASTB\). The server provides typing results (using in silico spoligotyping, large sequence polymorphisms, or specific variants) and predicts drug resistance to ciprofloxacin (CFX), ethambutol (EMB), INH, PZA, RMP, and streptomycin (SM) based on SNPs (Table 1)^13^. The results are stored online for seven days. The resistance prediction (R = resistant; <blank> = not resistant) includes no information about the evidence used in the analysis. We opened three browser tabs to analyse the fastq files of three samples in parallel, as suggested by the authors of the tool.

**Table 1:** Overview of the antibiotics and corresponding resistance genes analyzed by the different tools.

	First line drugs															Second line drugs									
Tool	INH					RMP	PZA		EMB				SM			AMK	CPR	KAN	FQ	CFX	OFX	MOX	ETH	LZD	PAS
	ahpC	inhA	kasA	katG	ndh	rpoB	pncA	rpsA	embA	embB	*embC*	*embR*	*gidB*	*rpsL*	*rss*										
CASTB	?	✓	?	✓	?	✓	✓	?	?	✓	?	?	?	✓	?					✓					
KvarQ		✓		✓		✓	✓	✓		✓				✓	✓	✓	✓	✓	✓						
Mykr. Pred. TB		✓		✓		✓				✓				✓	✓	✓	✓	✓	✓						
PhyResSE	✓	✓		✓	✓	✓	✓	✓	✓	✓	✓		✓	✓	✓	✓	✓	✓	✓				✓	✓	✓
TBProfiler	✓	✓	✓	✓		✓	✓	✓	✓	✓	✓	✓		✓	✓	✓	✓	✓	✓		✓	✓	✓		

Unless shown by a tick, resistance antibiotics or genes are not included. Question marks were used for CASTB where it was unclear whether the genes is interrogated, as no information regarding the rules underlying the interpretation is provided for this tool. We did not list resistance genes to second-line drugs as this was beyond the scope of this study, but more information for these drugs can be found in Supplementary Table S3.

#### KvarQ version 0.12.1

KvarQ is a software tool to scan fastq files for known variants^9^. The source code can be downloaded from GitHub (http://github.com/kvarq/kvarq/releases). Python and a C compiler are required to run the program. In the default setting, the user can choose between two so-called ‘testsuites’ for *M. tuberculosis*, which are collections of short sequences in a reference genome. The output is given in the ‘JavaScript Object Notation’ (json) format. The resistance catalogue includes variants located within 63 bp of *gyrA*, 81 bp of *rpoB*, 3 bp of *katG* and 561 bp of *pncA* (Supplementary Table S3). To analyse our WGS data, we had to merge our paired-end data for each strain into a single file. We used the default settings and standard MTBC testsuites to scan the fastq files. Finally, we produced a table that included all results using the summary-command, which is only available in the command-line version.

#### Mykrobe Predictor TB version 0.1.3

Mykrobe Predictor TB is designed for rapid antibiotic resistance prediction and species identification from WGS data of *M. tuberculosis*^10^. The tool and source-code can be downloaded via GitHub (https:\\github.com\iqbal-lab\Mykrobe-predictor\releases). The resistance panel consists of mutations in 62 codons; 27 in *rpoB*, one in *katG*, four in *inhA*, ten in *gyrA*, 14 in *rrs*, three in *embB*, two in *rpsL* and one in *eis* (Supplementary Table S3) to detect resistance against the drugs shown in Table 1. PZA is not included. Mykrobe Predictor TB is specifically designed to detect low-frequency resistance mutations. Therefore, Mykrobe Predictor TB reports even minor alleles at the positions included in the aforementioned list of resistance loci, but does not interpret them. We used the program with merged fastq files.

#### PhyResSE version 1.0

PhyResSE is a web-server for lineage identification and resistance prediction based on raw WGS data (www.phyresse.org)^12^. Its mutation catalogue is based on both a literature review and experimental data. The catalogue (Version 27) comprises 301 variants to predict resistance to 12 antibiotics (Table 1; Supplementary Table S3) and 239 SNPs in 135 genes for phylogenetic typing. We analysed our paired-end data using the batch mode and relied on the summary function to produce a table with the results for all strains.

#### TBProfiler

TBProfiler is a web-server that reports drug resistance and strain-type profiles (http:\\tbdr.lshtm.ac.uk\)^11^. Its output comprises resistance predictions for 11 antibiotics (Table 1) as well as information on the resistance mutation in question and further mutations in 22 candidate genes. Resistance predictions are based on a catalogue (including SNPs and indels) with 902 nucleotide positions at 26 loci that comprise six promotors and 20 coding regions (Supplementary Table S3). We ran the program offline after installing it locally (downloaded and installed 01\2016).

### Statistical analyses

Sensitivities and specificities, including the corresponding 95% confidence intervals, were calculated using the statistical software R (Version 3.1.0).

## Results

### Functionality

We compared CASTB, KvarQ, Mykrobe Predictor TB, PhyResSE and TBProfiler with regards to their handling, outputs, adjustability and ability to perform contamination checks. The full results of this comparison can be found in Table 2, but the following differences were notable.

**Table 2:** Overview of the features of the different analysis tools.

Feature	CASTB (Version 1.1)	KvarQ (Version 0.12.2)	Mykrobe Predictor TB (Version 0.1.3)	PhyResSE (Version 1.0)	TBProfiler
Web-based	Yes (registration needed)	No	No	Yes	Yes
Batchmode	No	Yes	In command-line version only	Yes	In command-line version only
Paired-end reads	Yes	Merged files only	Merged files only	Yes	Yes
Pipeline	Velvet de-Novo assembly; BIGSdb; MUMmer mapping; custom scripts	Python modules/packages with C extensions (no mapping)	Stampy mapping (H37Rv Version 2); variant calling SAMtools	BWA mapping(H37Rv Version 3), pre-processing & variant calling with GATK	snap mapping (selected regions of H37Rv version 3); variant calling SAMtools
Plain language report	Yes	No	Yes	Yes, detailed	Yes
Data export	Yes	Yes	Yes	Yes	No
Basis of lineage prediction	Virtual LSP, *in silico* spoligotyping, RFLP or MIRU-VNTR	Comas *et al*. 2009, Stucki *et al*. 2012 and unpublished	Stucki et al. 2012	Homolka *et al*. 2012, Coll *et al*. 2014 and other published	Coll *et al*. 2014
Detection of NTM	Yes	No (MTBC only)	Yes	No (MTBC only)	No (MTBC only)
Variants reported	None	Only resistance mutations	Only resistance mutations	All mutations	All mutations in candidate genes
Detection of mixed infections	Only Beijing-non Beijing	Yes	No	Yes	Yes
Hetero resistance	No	Visual	Yes, without quality scores	>10%	Yes
Modification possible	No	Yes	Yes	Yes	Yes

BIGSdb, Bacterial Isolate Genome Sequence database; BWA, Burrows-Wheeler Alignmet; GATK, Genome Analysis Toolkit; LSP, Large Sequence Polymorphism; MIRU-VNTR, Mycobacterial Interspersed Repetitive Units Variable Number of Tandem Repeats; NTM, Nontuberculous mycobacteria; RFLP, Restriction Fragment Length Polymorphism.

Only KvarQ and PhyResSE offer batch uploads, which meant that all 91 strains from our collection could be uploaded in parallel and processed automatically. The other tools either have no batch mode at all (CASTB, TBProfiler) or can only analyse files in batches in the command-line version (Mykrobe Predictor TB). Moreover, paired-end fastq files have to be merged for KvarQ and Mykrobe Predictor TB, which may represent a challenge for some users.

The ability to export and store the outputs differed significantly between the tools. In the case of PhyResSE, the complete reports for all strains analysed in the same session can be exported easily as a ‘comma separated values’ (csv) file. Similar ‘batch mode’ reports were not available for CASTB or limited to the command-line version of KvarQ. Customised perl scripts were required to obtain comparable reports for Mykrobe Predictor TB and TBProfiler. The results of single strains can be stored in the json format for Mykrobe Predictor TB and KvarQ, whereas PhyResSE and CASTB used the csv format. By contrast, no dedicated export and storage function exists for TBProfiler.

A further important difference between the tools was the ease in which they could be updated. Specifically, it is important to refine the list of resistance variants whenever the understanding of the genetic basis of antibiotic resistance improves. Currently, only PhyResSE allows users to extend the existing list of resistance mutations by simply updating a downloadable list of variants. For CASTB no adjustments are possible, whereas for the remaining tools more advanced skills are needed. In the case of TBProfiler, adjustments are possible by editing the source code. The testsuites, which are Python source files, can be changed for KvarQ. A code generator in Python is provided to modify the variant list of Mykrobe Predictor TB.

### Phylogenetic lineage classification

Each tool differs in its approach for epidemiological typing. CASTB is the only tool that interrogates large sequence polymorphisms, whereas all other tools rely exclusively on SNPs, but use different SNP catalogues. KvarQ combines SNPs from Comas *et al*. and Stucki *et al*.^17^, whereas Mykrobe Predictor TB only uses the latter study^18,19^. The SNP catalogues from Homolka *et al*. and Coll *et al*. form the basis of the classification in PhyResSE^16,20^. TBProfiler only employs the scheme from Coll *et al*.^20^ Despite these varied approaches, the results for 73 strains only differed in their resolution (i.e. tools that rely on Coll *et al*., which represents the most advanced typing scheme to date, provided the best resolution at the sub-lineage level)^20^. All tools classified the 11 strains, for which our classical experimental techniques had failed to provide a result (Table 3).

**Table 3:** Lineage prediction of the various tools compared with traditional typing methods.

Genotype	#strains	CASTB	KvarQ	Mykrobe Predictor TB	PhyResSE	TBProfiler
EAI	4	lineage 1 (Indo-Oceanic lineage)	lineage 1	East African/Indian ocean	EAI/lineage1.1.1	lineage1.1.1
Beijing	4	lineage 2 (East-Asian lineage)	lineage 2/beijing sublineage	Beijing/East Asia	Beijing/lineage2.2.1	lineage2.2.1
Ghana	1	lineage 4 (Euro-American lineage)	lineage 4	European/American	Ghana/lineage4.1	lineage4.1
Sierra Leone-1	7	lineage 4 (Euro-American lineage)	lineage 4	European/American	Euro-American Superlineage/lineage4.1	lineage4.1
X-type	1	lineage 4 (Euro-American lineage)	lineage 4	European/American	Euro-American Superlineage/lineage4.1.1.1	lineage4.1.1.1
	1	lineage 4 (Euro-American lineage)	lineage 4	European/American	Euro-American Superlineage/lineage4.1.1.3	lineage4.1.1.3
Haarlem	12	lineage 4 (Euro-American lineage)	lineage 4	European/American	Haarlem/lineage4.1.2.1	lineage4.1.2.1
LAM	9	lineage 4 (Euro-American lineage)	lineage 4	European/American	LAM/lineage4.3.3	lineage4.3.3
	1	**lineage*M. bovis***	lineage 4	European/American	LAM/lineage4.3.3	lineage4.3.3
	2	lineage 4 (Euro-American lineage)	lineage 4	European/American	LAM/lineage4.3.4.1	lineage4.3.4.1
	3	lineage 4 (Euro-American lineage)	lineage 4	European/American	LAM/lineage4.3.4.2	lineage4.3.4.2
S-type	3	lineage 4 (Euro-American lineage)	lineage 4	European/American	S-type/lineage4.4.1.1	lineage4.4.1.1
Cameroon	4	lineage 4 (Euro-American lineage)	lineage 4	European/American	Cameroon/lineage4.6.2.2	lineage4.6.2.2
Sierra Leone-2	9	lineage 4 (Euro-American lineage)	lineage 4	European/American	Euro-American Superlineage/lineage4.8	lineage4.8
West African 1	6	lineage 5 (West-African lineage 1)	lineage 5	**Unknown Species**	West African 1/lineage5	lineage5
West African 2	14	lineage 6 (West-African lineage 2	lineage 6	*M. africanum*	West African 2/lineage6/BOV_AFRI	West African 2/6/BOV_AFRI
	3	lineage 4 (Euro-American lineage)	lineage 4	European/American	Euro-American Superlineage/lineage4.1	lineage4.1
	3	lineage 4 (Euro-American lineage)	lineage 4	European/American	Euro-American Superlineage/lineage4.1.1.3	lineage4.1.1.3
None	1	lineage 4 (Euro-American lineage)	lineage 4	European/American	Haarlem/lineage4.1.2.1	lineage4.1.2.1
	1	lineage 4 (Euro-American lineage)	lineage 4	European/American	Euro-American Superlineage/lineage4.6	lineage4.6.1
	3	lineage 4 (Euro-American lineage)	lineage 4	European/American	Euro-American Superlineage/lineage4.8	lineage4.8

CASTB classified one 4.3.3/LAM strain as *M. bovis*, whereas Mykrobe Predictor TB was unable to classify strains belonging to lineage 5/West African lineage 1, as shown in bold.

Of the remaining seven discrepancies, six might have affected contact tracing only (i.e. all six *M. africanum* West African 1 strains were reported as ‘unknown species’ by Mykrobe Predictor TB). By contrast, the misclassification of the *M. tuberculosis* Latin-American Mediterranean (LAM) strain 10205-03 (ERS457959) as *M. bovis* by CASTB might have resulted in a major diagnostic error if PZA resistance had been inferred, given that *M. bovis* is intrinsically resistant to this antibiotic^14,21^. The strain was in fact phenotypically susceptible and did not harbor any *pncA* mutations (Supplementary Table S3). Notably, it was not reported as PZA resistant by CASTB. However, in light of the fact that CASTB does not provide a list of mutations used to infer resistance, it would be reasonable for a user of the tool to assume that the mutation responsible for intrinsic resistance in *M. bovis (pncA* H57D) was missing from the catalogue, which would explain the apparent susceptible result based on *pncA* (the mutation does, indeed, appear not to feature in the catalogue given that our three *M. bovis* strains were not reported as PZA resistant by the tool)^14^.

As a consequence, KvarQ, PhyResSE and TBProfiler showed an accuracy of 100% for lineage identification, whereas the accuracy of CASTB and Mykrobe Predictor TB were 98.9% and 93.4%, respectively.

### Resistance prediction

The sensitivities and specificities for predicting resistance compared with phenotypic DST varied significantly between the different tools and antibiotics (Table 4). Whereas sensitivities and specificities for INH and RMP were high for all tools evaluated, this was much more variable for the other first-line drugs. In fact, the sensitivities for four tool-drug combinations ranged between just 22–44% (Table 4). The equivalent values with Sanger sequencing as the reference standard can be found in Supplementary Table S4.

**Table 4:** Sensitivities and specificities of the WGS tools to predict resistance to first-line drugs compared with phenotypic DST.

Drug	DST		CASTB		KvarQ		Mykr. Pred. TB		PhyResSE		TBProfiler	
	#R	#S	Sens	Spec	Sens	Spec	Sens	Spec	Sens	Spec	Sens	Spec
INH	29	62	0.83 (0.64, 0.94)	0.98 (0.91, 1.00)	0.79 (0.60, 0.92)	0.98 (0.91, 1.00)	0.79 (0.60, 0.92)^1^	0.95 (0.87, 0.99)	0.93 (0.77, 0.99)	0.97 (0.89, 1.00)^2^	0.90 (0.73, 0.98)	0.84 (0.72, 0.92)^2^
RMP	14	77	1.00 (0.68, 1.00)	0.96 (0.89, 0.99)	0.93 (0.66, 1.00)	0.95 (0.87, 0.99)	1.00 (0.68, 1.00)	0.94 (0.85, 0.98)^2,3^	1.00 (0.68, 1.00)	0.94 (0.85, 0.98)	1.00 (0.68, 1.00)	0.94 (0.85, 0.98)
SM	37	54	0.30 (0.16, 0.47)	1.00 (0.90, 1.00)	0.57 (0.39, 0.73)	1.00 (0.90, 1.00)	0.57 (0.39, 0.73)	1.00 (0.90, 1.00)	0.84 (0.68, 0.94)	0.98 (0.90, 1.00)	0.57 (0.39, 0.73)	1.00 (0.90, 1.00)
EMB	14	77	0.57 (0.29, 0.82)	1.00 (0.93, 1.00)	0.50 (0.23, 0.77)	0.99 (0.93, 1.00)	0.50 (0.23, 0.77)^3^	0.99 (0.93, 1.00)	0.86 (0.57, 0.98)	0.97 (0.91, 1.00)	0.86 (0.57, 0.98)	0.96 (0.89, 0.99)
PZA	9	82	0.44 (0.14, 0.79)	0.99 (0.93, 1.00)	0.22 (0.03, 0.60)	0.96 (0.90, 0.99)	n.a.	n.a.	0.67 (0.30, 0.93)	0.96 (0.90, 0.99)	0.44 (0.14, 0.79)	0.96 (0.90, 0.99)

The numbers in parentheses represent 95% confidence intervals. The equivalent table with Sanger sequencing as comparator can be found in Supplementary Table S4.

INH, isoniazid; RMP, rifampicin; SM, streptomycin; EMB, ethambutol; PZA, pyrazinamid; R, resistant; S, susceptible; sens, sensitivity; spec, specificity; n.a., not applicable.

^1^Mutations that were merely reported but not interpreted were excluded from the analysis.

^2^*kasA* and *inhA* were not Sanger sequenced for some strains.

^3^Low-frequency variants that were only observed upon re-examination of the Sanger sequences were treated as wild-type since these mutations would not have been called unless the WGS data had been available.

Incorrect resistance predictions can either arise because of four factors (see discussion for more details):

a The inadequate limit of detection of the genotypic method,
b Errors in variant calling,
c Incorrect interpretation of variants (using the mutation catalogues in Supplementary Table S3), or
d Errors in phenotypic DST.

#### CASTB

In the case of CASTB, we were unable to dissect these sources of error since this tool does not provide an output of the variants identified in a particular sample. Moreover, the authors of this tool have not included a list of mutations that are used to predict resistance (the genes shown in Table 1 were inferred based on the analyses for this study). As a result, we were only able to conclude that 61 out of 66 resistance calls by this tool were valid compared with phenotypic DST, but could not assess whether these predictions were based on the correct mutations, as opposed to another mutation that happened to occur in the strains in question. However, 41 cases of resistance were missed compared with phenotypic DST.

#### Differences in variant calling

Differences in variant calling in regions, for which Sanger data were available, were observed in 11 cases (Supplementary Table S2). In eight of those cases, involving one *katG* and seven *rpoB* mutations, Mykrobe Predictor TB reported minor mutations that did not meet the threshold to be reported as causing resistance. These occurred in strains that were INH and RMP susceptible, respectively. The corresponding Sanger results did not support the presence of minor resistance mutations. We investigated this further by examining the Stampy-mapped WGS data with the Integrative Genome Viewer. This showed that the majority of mutated bases had low quality scores (below 15, i.e. the probability of being called incorrectly is above 3%), which suggested that these were likely sequencing errors (Supplementary Table S2 and Figure S1). Because these mutations were detected, but not reported to cause resistance, we did not regard these as errors when calculating the sensitivities and specificities of the WGS analysis tools (Table 4 and Supplementary Table S4).

Heteroresistance was the cause for differences in variant calling in three cases. The *rpoB* L452P and *embB* M306I mutations, which were reported by at least one WGS tool, were originally missed by Sanger sequencing in strains 8082-03 and 8867-03, respectively. A re-examination of the electropherograms for both strains confirmed the presence of these mutations (data not shown). Based on an inspection of the mapped WGS data, strain 10517-03 contained at least two *rpoB* populations. The T400A and S450L mutations occurred in the same population at approximately 65% of the total. The H445Y occurred at about 21% and was initially missed by Sanger sequencing. PhyResSE and TBProfiler reported both populations. A third potential population with an S441L mutation, which was not detectable using Sanger sequencing as it had a frequency of just 14%, was reported by Mykrobe Predictor TB as a low-quality call and was consequently not interpreted (Z. Iqbal, personal communication). Mykrobe Predictor TB did not report the S450L mutation despite the fact that, unlike the T400A mutation, S450L is listed of its resistance catalogue (Supplementary Table S3). This was due to a bug in the software, which is due to be corrected (Z. Iqbal, personal communication).

#### Differences in interpretation

Differences in interpretation of mutations, for which Sanger data were available, accounted for most differences in resistance predictions. Specifically, 52 mutations were not reported by at least one WGS analysis tool because they do not feature in the respective lists that each tool relies on for the interpretation of variants (10 for PhyResSE, 35 for KvarQ, 40 for Mykrobe Predictor TB, and 24 for TBProfiler, as shown in Table 5). It was unclear why the *embB* G406D mutation in strain 1599-04 was not reported by Mykrobe Predictor TB despite featuring in the resistance catalogue of this tool. This issue has been corrected in a recently released version of the tool.

**Table 5:**
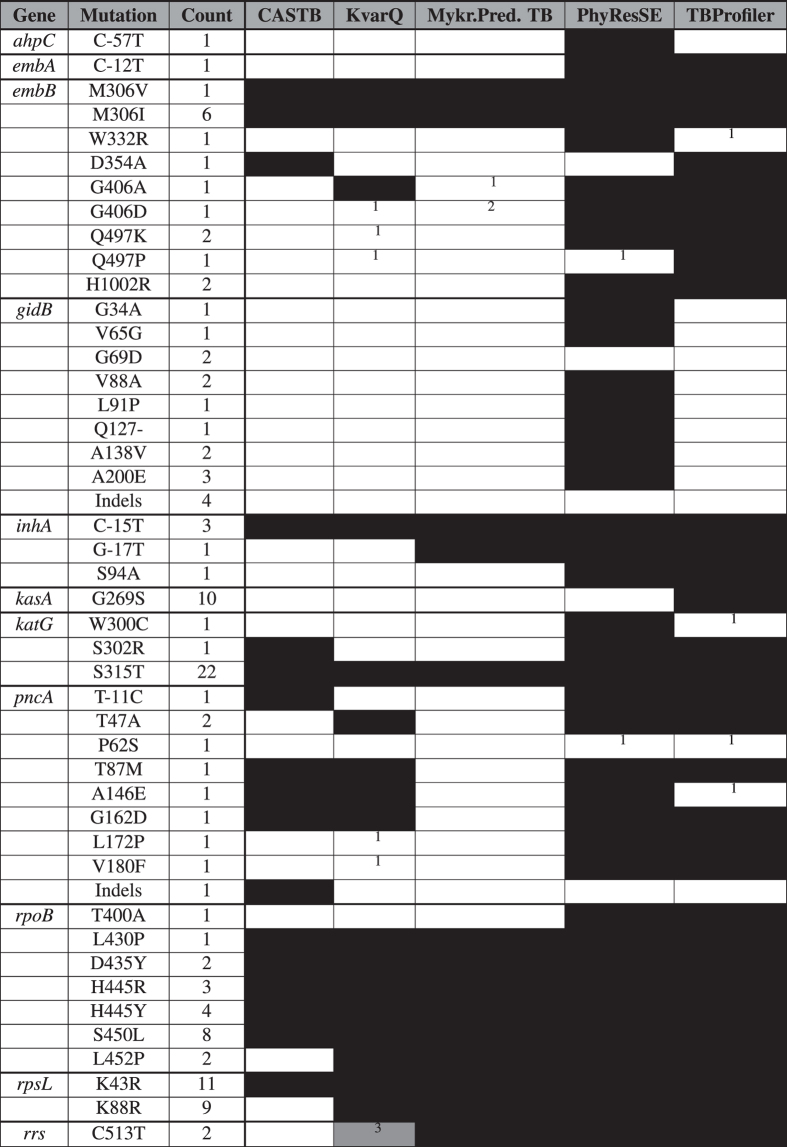
Overview of the resistance mutations detected and interpreted by Sanger sequencing compared with WGS tools.

Black boxes show if a mutation was called and interpreted as causing resistance by a particular WGS analysis tool based on their respective mutation catalogues (Supplementary Table S3). For CASTB, the black boxes corresponded to a resistance call, which were included for completeness, although it was unclear whether the mutations in questions were actually the ones that were interpreted since the tool does not provide a mutation catalogue. The numbers in parentheses represent 95% confidence intervals. The equivalent table with Sanger sequencing as comparator can be found in Supplementary Table S4. ^1^Only a nucleotide change that resulted in a different amino acid change at that position was included in the resistance catalogue of this tool. ^2^It was unclear why this mutation, which featured in the resistance catalogue of this tool, was not reported. ^3^Reported as S171S (i.e. the mutation was translated despite the fact that it was within the 16S rRNA region).

#### Other cases

There were 18 cases in which resistance mutations were reported by at least one tool, for which no Sanger data were available (Supplementary Table S5). All of these mutations were called correctly based on an inspection of the WGS data. In six of these cases (strains 3156-04, 4139-04, 8864-03, 10011-03, 2509-04, and 7520-04) this did not make a difference for predicting INH resistance given that they coincided with INH resistance mutations elsewhere in the genome that were interpreted. In two strains the detection of these additional mutations increases the sensitivity compared with phenotypic DST (4518-03 and 8868-03)^22,23^.

For seven strains, TBProfiler inferred INH resistance based on the *kasA* G269S mutation, resulting in false-resistant results. However, allelic exchange experiments have demonstrated that this mutation does not confer INH resistance^23^. In accordance with this observation, the authors of TBProfiler had recognised that this was merely a phylogenetic marker for a subgroup of the LAM family^11,20^. Yet, contrary to Supplementary Table 2 in Coll *et al*., in which they claim to have excluded this mutation, *kasA* G269S features in their resistance catalogue (Supplementary Table S3), which accounted for the incorrect interpretation of this mutation^11^.

Finally, TBProfiler reported 12657-03 and 11096-03 to be resistant to INH and EMB because of *ahpC* C-81T and *embB* Q497P mutations, respectively. The role of both mutations is poorly understood and both strains tested susceptible to the respective drugs (Supplementary Table S5).

#### False-resistance compared with phenotypic DST

In total 23 strains were reported as resistant by at least one genotypic method. In addition to the strains discussed above, two cases of false-resistance to EMB, two to INH, four to PZA, five to RMP, and one to SM were observed (Supplementary Table S6).

#### False-susceptibility compared with phenotypic DST

There were three resistances that were missed by Sanger as well as all WGS analysis tools (Supplementary Table S6).

## Discussion

In this study, we performed the most comprehensive comparison of five software pipelines for the automated analysis and interpretation of WGS data of MTBC strains to date. Unlike more general WGS analysis pipelines, the ultimate goal for these tools is to be used clinically, as opposed to research. Consequently, their DST results based on either the species identification or the detection and interpretation of resistance variants need to be highly accurate and standardized.

We had expected that false-susceptible results would occur because of differences in variant calling of heteroresistant strains (i.e. phenotypic DST can detect resistance at 1% (or 10% in the case of PZA), whereas the limit of detection of WGS depends on the sequence coverage^24^) and because of resistance mutations that did not feature in the catalogues used for the interpretation of variants, as shown by Phelan *et al*., who recently compared TBProfiler and Mykrobe Predictor TB using 10 clinical strains^15^. Specifically, we found that differences in interpretation mainly affected EMB, PZA, and SM, for which the genetic basis of resistance is more complex than for RMP and INH.

Yet, we had not expected false-resistance calls because of the inappropriate interpretation of known polymorphisms that do not cause resistance, as was the case with TBProfiler and the *kasA* G269S mutation with regards to INH, a key drug for the treatment of TB (Supplementary Table S6)^14^. Notably, this flaw appeared to be specific to the command-line version of TBProfiler, whereas the online version interpreted this mutation correctly (data not shown). In a separate study, we found an additional example in which KvarQ, Mykrobe Predictor TB, and TBProfiler generated false-resistance results to fluoroquinolones with the *gyrA* T80A+A90G double mutations^25^. This underlines the importance of developing a clinical-grade database to interpret resistance mutations, as is currently underway with the ReSeqTB initiative^14,26^. However, it has to be appreciated that new resistance mutations will continue to be discovered over time, particularly in non-essential genes where the spectrum of resistance mutations is large^6^. Therefore, genotypic assays, including WGS, should generally only be used as tools to rule-in resistance and phenotypic DST will still be needed to confirm susceptibility^4^.

By contrast, the remaining 15 cases of false-susceptibility were likely mostly due to problems with phenotype rather than the genotypic results (Supplementary Table S6). For example, mutations in *embB* are known to result in only slight MIC increases to EMB, which means that the MIC distributions of wild-type and mutated strains overlap, unless secondary mutations increase the MICs even further^27,28,29,30^. Similarly, several *rpoB* mutations are known to test susceptible in MGIT, but resistant on Löwenstein-Jensen medium^31,32,33^. These false-susceptible results with MGIT are likely due to a breakpoint artefact (i.e. because the critical concentration is set above the epidemiological cut-off)^4,34,35^. A thorough re-evaluation of the critical concentrations using modern principles is the only option to resolve these systematic differences^4,24,36^.

We also had not anticipated to find a case in which the misidentification of an *M. tuberculosis* strain as *M. bovis* by CASTB would result in a false-resistance result to PZA, if this species identification was used as a surrogate for intrinsic resistance to this drug^14^. Importantly, we were not able to investigate what the reason for this error was, since CASTB takes automation to an extreme by providing very little information on how it works and no evidence at all about variants detected in a particular strain that were used to infer resistance or the lineage. In our view, tools that function as complete ‘black boxes’ should not be used clinically since their independent assessment is only possible by trial and error (e.g. we actually had to analyse three *M. bovis* strains to ascertain that the *pncA* H57D does not feature in its resistance catalogue).

From the point of view of addressing the discrepancies identified in this study, the errors fall into two groups. Both false-positives and false-negative results due to incorrect variant interpretation can be solved by simply updating the resistance catalogues, although, in practice, this is easier for some tools than others, as outlined in the ‘functionality’ section above. By contrast, differences in variant calling and the ease of use of the tools would require more extensive modifications. This includes improved algorithms for the detection of resistance caused by insertions and deletions, particularly in heteroresistant strains^4,24,37^. In this context, we would recommend for Mykrobe Predictor TB not to report low-quality, low-frequency mutations even if these are not interpreted. In our view, software tools should only report high-quality mutations in order not to risk that inexperienced users reach wrong conclusions (e.g. it was not clear from the output for 10517-03 which *rpoB* mutation the tool had used to infer RMP resistance).

This raises a more fundamental question. We believe that the central issue is not which tool is currently ‘best’, but how these tools will be maintained and improved. The recent interest by academics in translating WGS, which has resulted in the development of the tools investigated in this study, is a welcome development. However, none of the tools currently combines all features needed to meet regulatory requirements, such as record-keeping capabilities and version control^38^. Even if they did, academics would be unlikely to maintain and refine these tools (e.g. to adjust them to solve yet unknown reasons for incorrect variant calling or to accommodate novel sequencing technologies). Instead, viable models are called for that ensure the longevity of analysis tools, whilst preventing private monopolies, as has been the case with genetic testing in other areas^39^.

## Additional Information

**How to cite this article:** Schleusener, V. *et al. Mycobacterium tuberculosis* resistance prediction and lineage classification from genome sequencing: comparison of automated analysis tools. *Sci. Rep.* **7**, 46327; doi: 10.1038/srep46327 (2017).

**Publisher's note:** Springer Nature remains neutral with regard to jurisdictional claims in published maps and institutional affiliations.

## Supplementary information

Supplementary Figure S1 (PDF 541 kb)

Supplementary Dataset S1 (XLS 31 kb)

Supplementary Dataset S2 (XLS 100 kb)

Supplementary Dataset S3 (XLS 244 kb)

Supplementary Dataset S4 (XLS 61 kb)

Supplementary Dataset S5 (XLS 33 kb)

Supplementary Dataset S6 (XLS 35 kb)
