# Supplementary material for: Mycobacterium tuberculosis resistance prediction and lineage classification from genome sequencing: comparison of automated analysis tools
Source: Sci Rep. 2017 Apr 20;7:46327. doi: 10.1038/srep46327 (PMC7365310; doi:10.1038/srep46327)

Mycobacterium tuberculosis resistance prediction and lineage classification from genome sequencing: comparison of automated analysis tools

Viola Schleusener[1], Claudio U. Köser[2], Patrick Beckert[1, 3], Stefan Niemann[1, 3*], and Silke Feuerriegel[1, 3*]

[1] Division of Molecular and Experimental Mycobacteriology Group, Research Center Borstel, Borstel, Germany

[2] Department of Medicine, University of Cambridge, Cambridge, United Kingdom

[3] German Center for Infection Research, Borstel Site, Borstel, Germany

Corresponding author: Stefan Niemann, Division of Molecular and Experimental Mycobacteriology Group, Research Center Borstel, Borstel, Germany. Tel: 0049-4537-1887620, E-Mail: sniemann@fz-borstel.de

[*]SN and SF contributed equally to this work

Supplementary Figure S1.

Mapping results (stampy) for Mykrobe Predictor TB's low-frequency variants in *rpoB* (n=7) and *katG* (n=1)

IGV mapping of 1327-04 at genome position 2155168

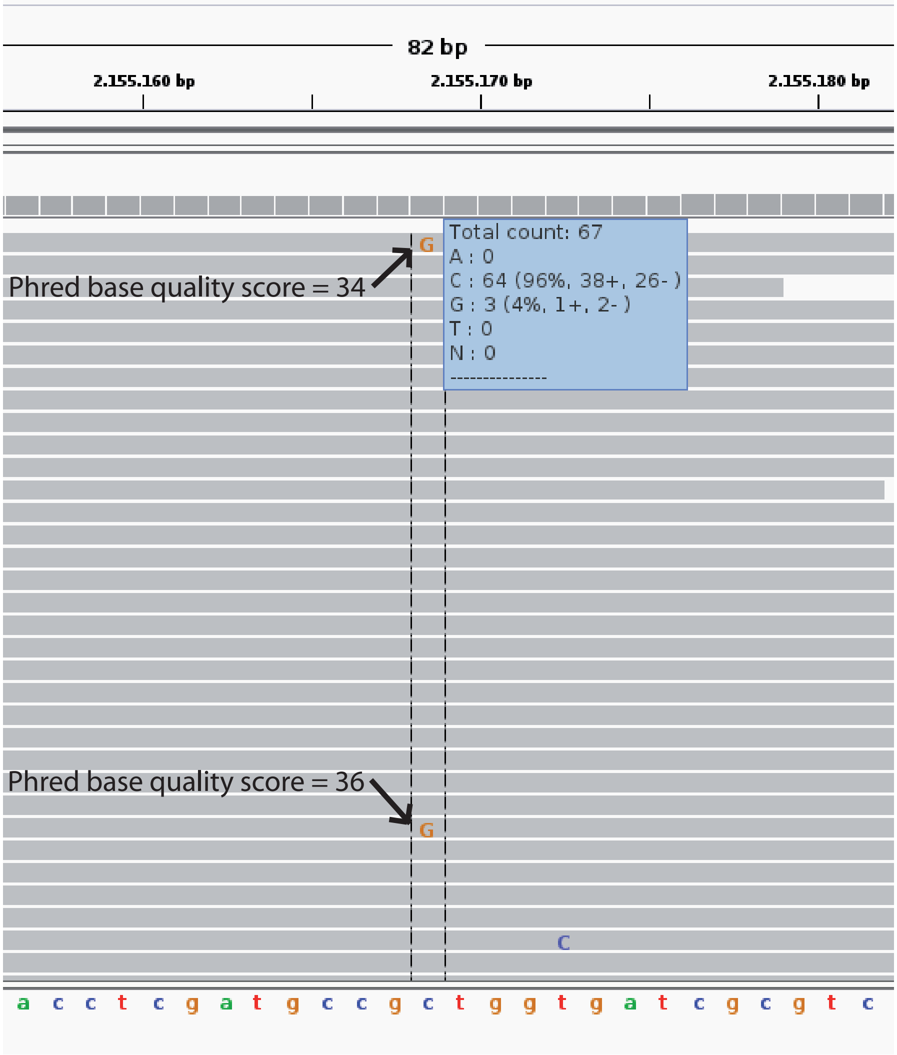

IGV mapping of 3158-04 at genome position 761154

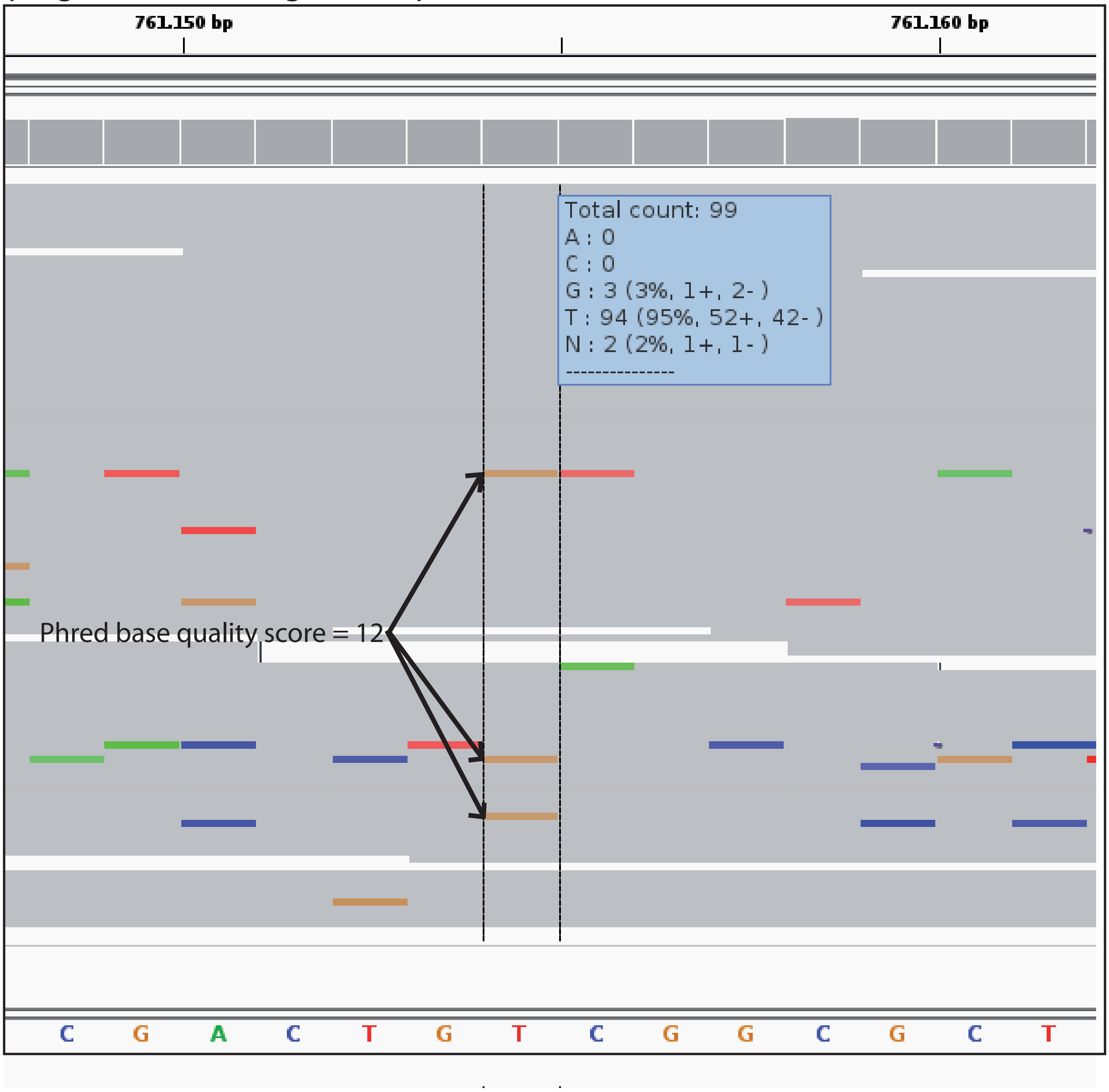

IGV mapping of 4712-04 at genome position 761079

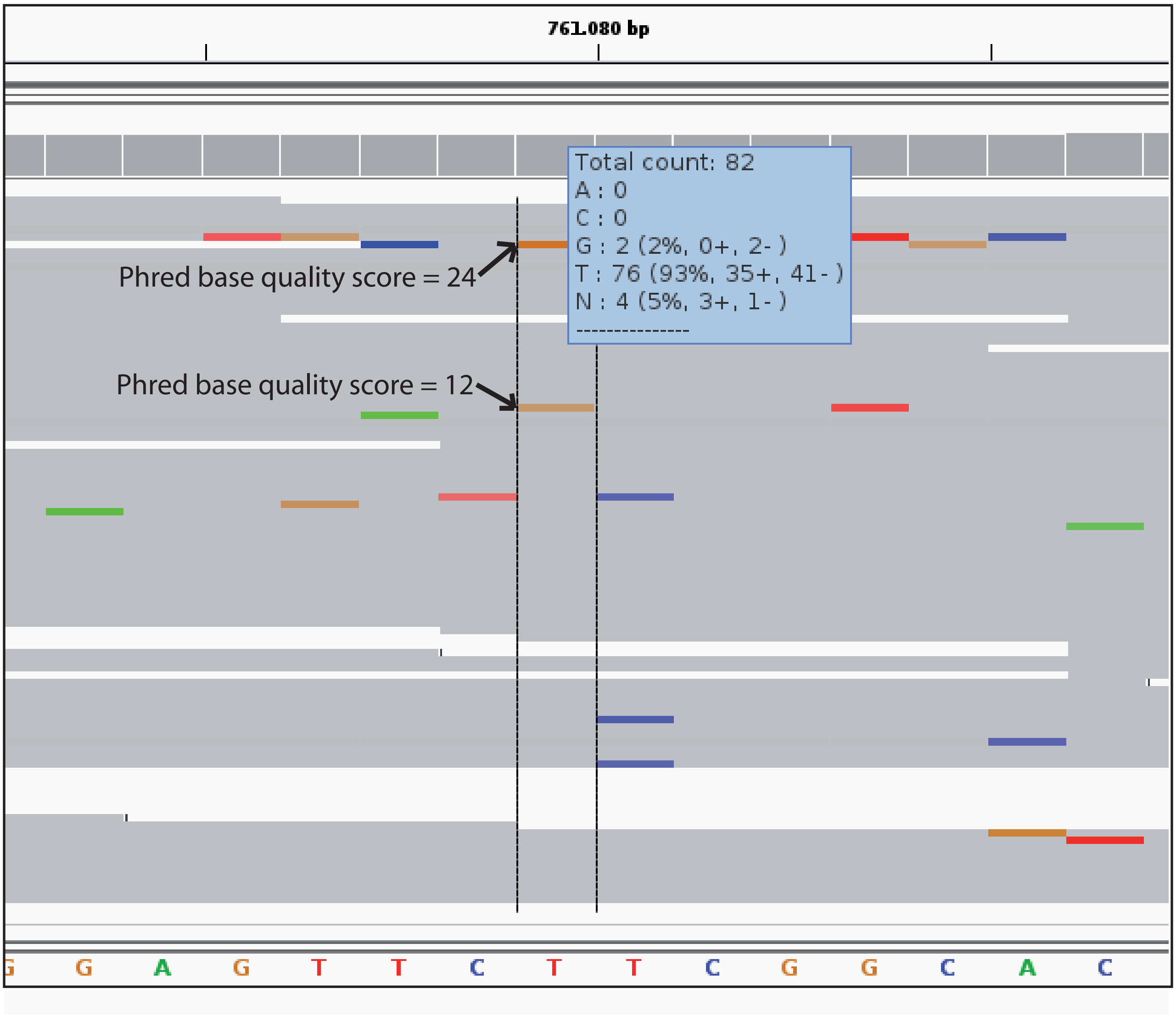

IGV mapping of 4717-04 at genome position 761139

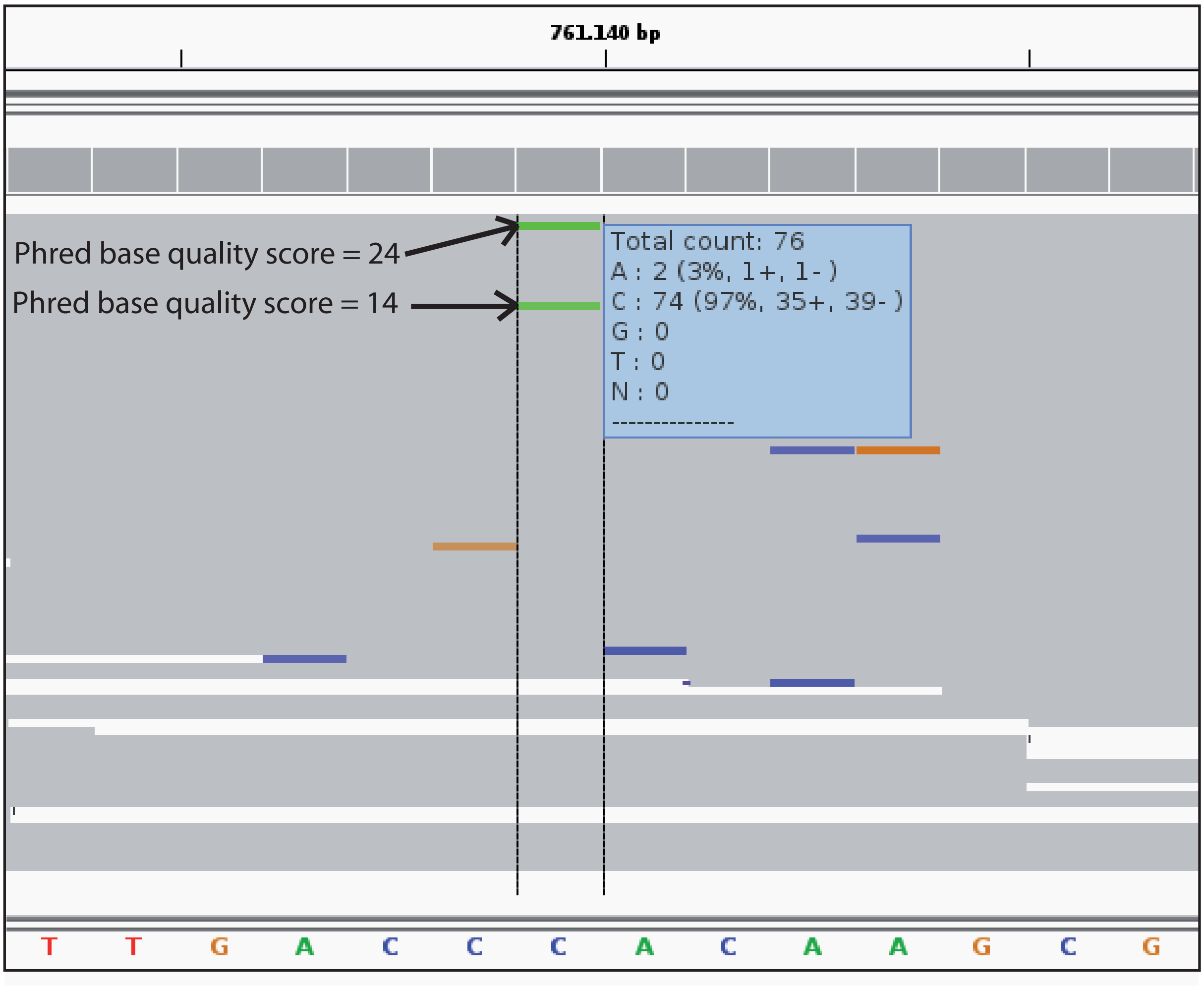

IGV mapping of 6640-04 at genome position 761154

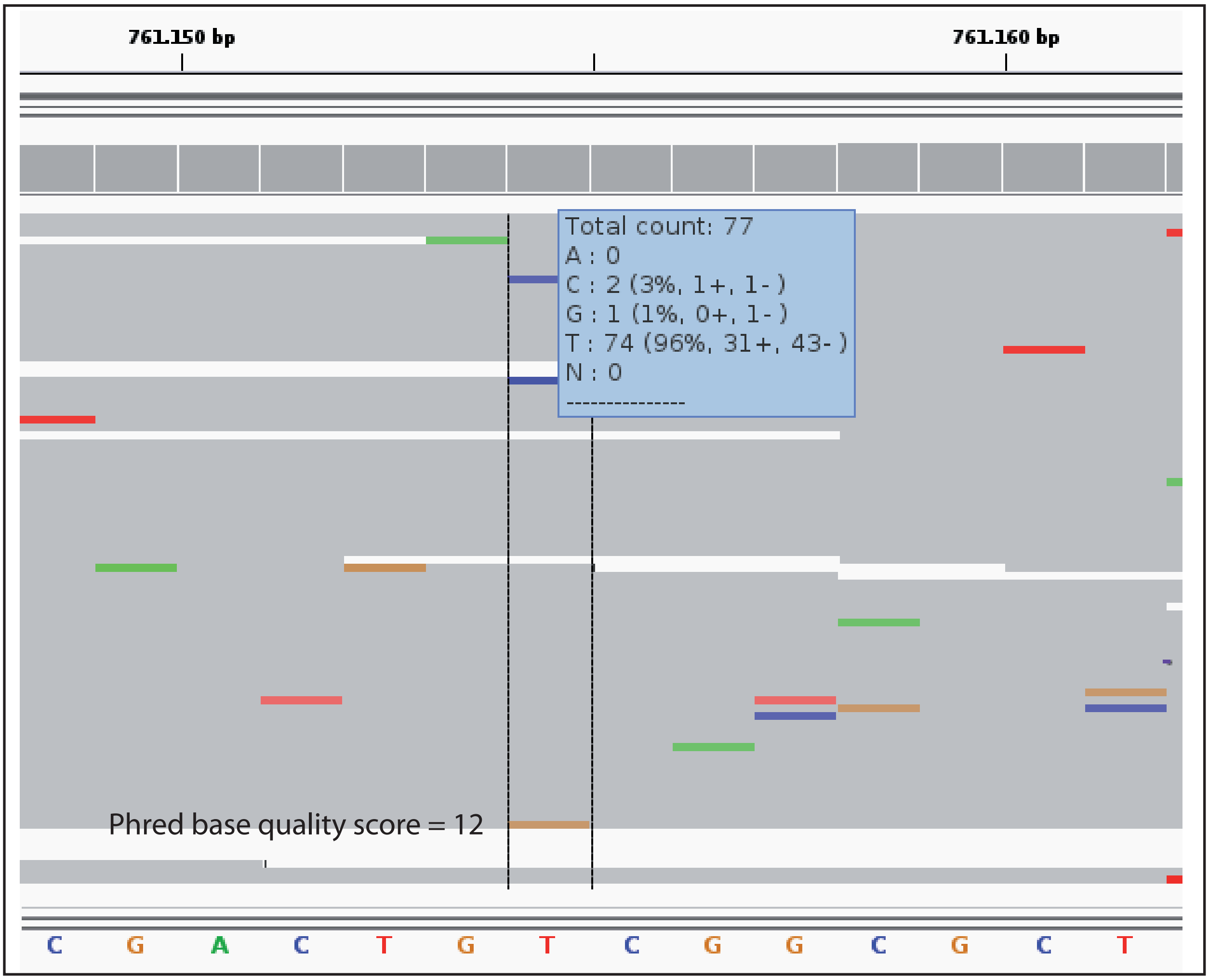

IGV mapping of 8082-03 at genome position 761133

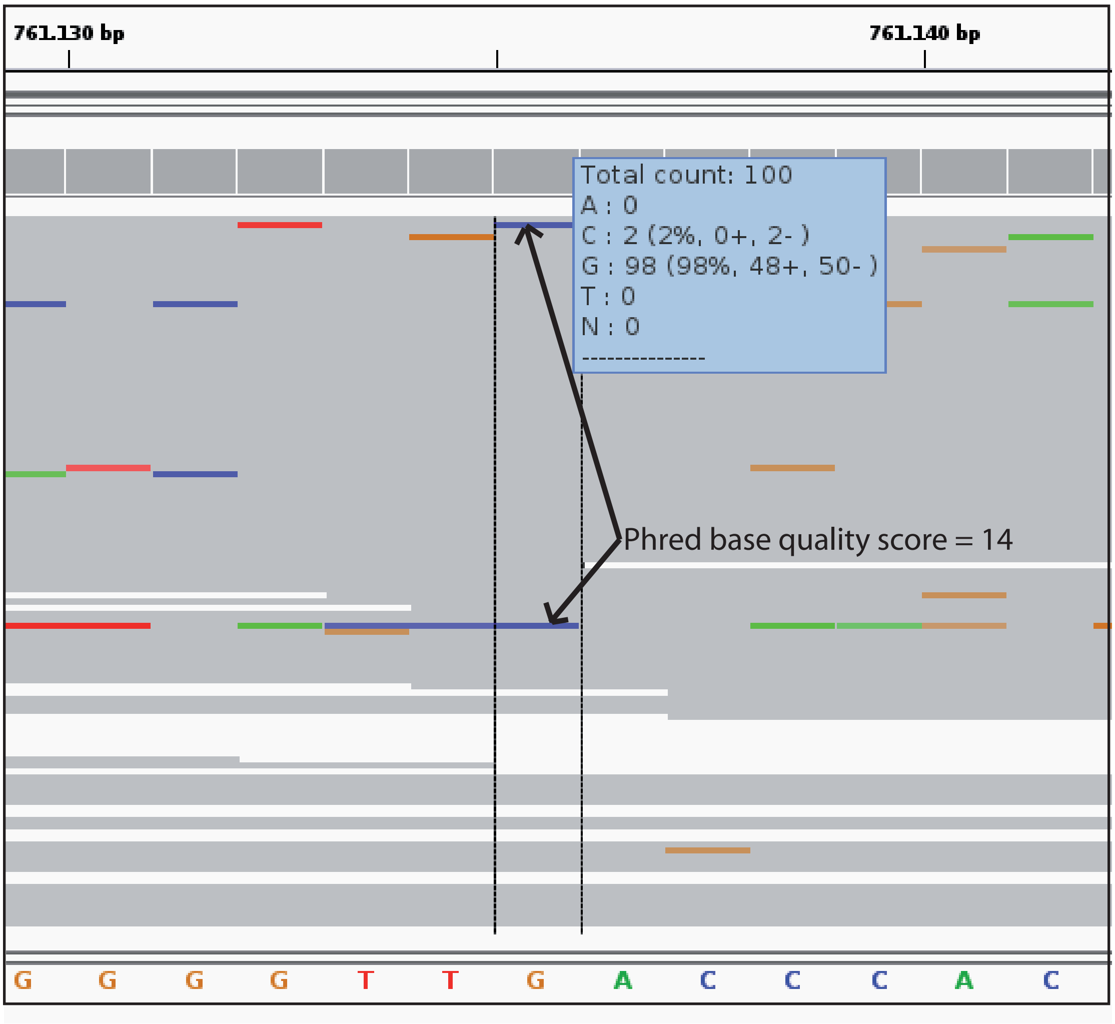

IGV mapping of 10205-03 at genome position 761133

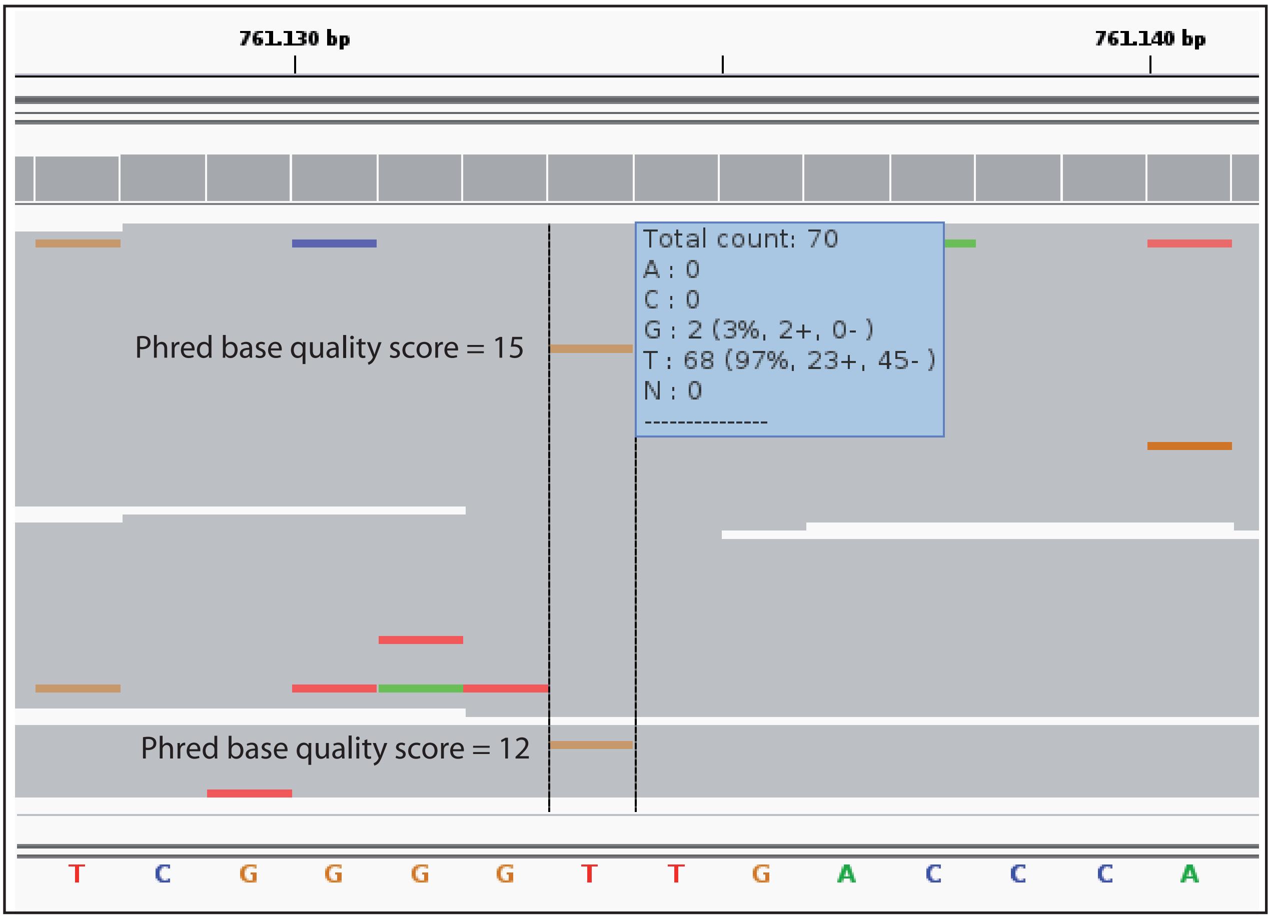

IGV mapping of 11818-03 at genome position 761108

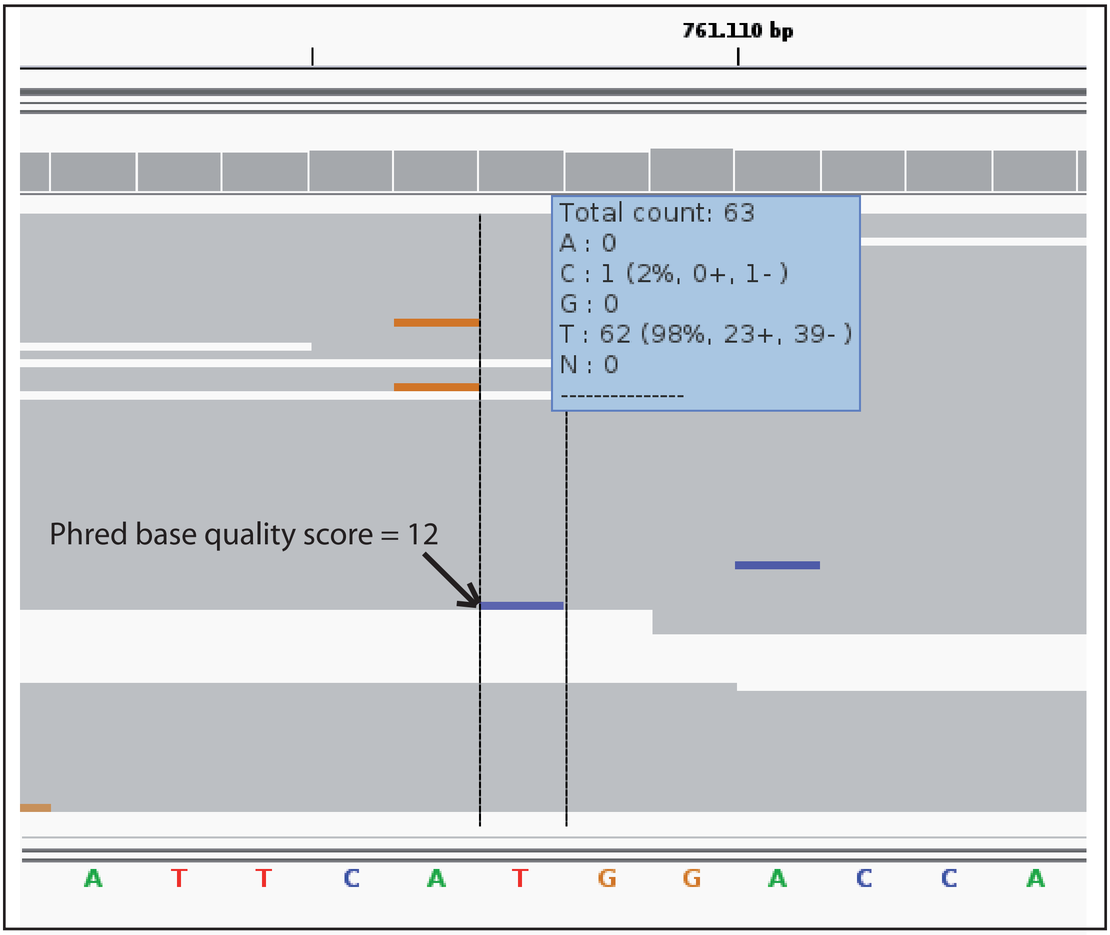

Supplement: Supplementary file 1 — Supplementary Figure S1 (PDF 541 kb) [file 41598_2017_BFsrep46327_MOESM18_ESM.pdf]
